# Impact of surgeon learning curve in minimally invasive radical hysterectomy on early stage cervical cancer patient survival

**DOI:** 10.52054/FVVO.13.3.035

**Published:** 2021-09-24

**Authors:** L Pedone Anchora, N Bizzarri, V Gallotta, V Chiantera, F Fanfani, A Fagotti, F Cosentino, G Vizzielli, V Carbone, G Ferrandina, G Scambia

**Affiliations:** Fondazione Policlinico Universitario A. Gemelli, IRCCS, UOC Ginecologia Oncologica, Dipartimento per la salute della Donna e del Bambino e della Salute Pubblica, Roma, Italy; Department of Gynecologic Oncology, University of Palermo, Palermo, Italy; Università Cattolica del Sacro Cuore, Istituto di Ginecologia e Ostetricia, Roma, Italy; Gemelli Molise, Dipartimento di Oncologia, UOC Ginecologia Oncologica, Campobasso, Italy

**Keywords:** cervical cancer, minimally invasive, radical hysterectomy, experience, learning curve, laparoscopy

## Abstract

**Background:**

Recently, it has been sustained that only surgeons skilled in minimally invasive radical hysterectomy (MI-RH) could provide valuable oncological outcomes in early-stage cervical cancer. Still, literature lacks data correlating surgeon experience with patient survival rate.

**Objective:**

To investigate the impact of surgeon training patient survival rate following MI-RH for early stage cervical cancer.

**Methods:**

This was a retrospective study of 243 early-stage cervical cancer treated with MI-RH. Multiple regression analyses were undertaken to investigate the impact of the surgeons learning curve, according to the number of MI-RH, on patients prognosis.

**Results:**

A steady trend of reduction in disease recurrence risk is associated with increased surgeon experience. The peak of the learning curve was shown at the 19th MI-RH (hazard ratio of disease-free survival: 0.321; 95%CI: 0.140-0.737; p= 0.007). The 3 years disease-free survival that a surgeon could provide to patients is significantly lower at the beginning of his/her learning path comparing to what he/she could guarantee once adequate experience had been achieved (75.4% and 91.6% respectively, p=0.005). Surgeon experience appears to be an independent prognostic factor.

**Conclusion:**

The experience that a surgeon can achieve practicing in MI-RH significantly influences oncological outcomes of early-stage cervical cancer patients. Future studies comparing minimally invasive and open surgery should take this into account. It would be advisable that the scientific community precisely establishes the minimum training required in the field of MI-RH for early-stage cervical cancer.

## Background

Radical hysterectomy with pelvic lymphadenectomy is the main treatment for women suffering from early-stage cervical cancer (Bansal et al., 2009; National Comprehensive Cancer Network Cervical Cancer Guidelines, 2019) and has been traditionally carried out by open radical surgery through a laparotomy incision.

During the last decades, minimally invasive radical hysterectomy (MI-RH) for early-stage cervical cancer patients became increasingly popular on the basis of several retrospective studies (Diver et al., 2017; Nam et al., 2012; Corrado et al., 2018; Laterza et al., 2016; Gallotta et al., 2018; Barletta et al., 2015) and two meta-analyses reporting equivalent oncological outcomes between MI-RH and open radical hysterectomy (O-RH), in the face of reduced intra- and post-operative complications, estimated blood loss, and hospitalisation time (Diver et al., 2017; Nam et al., 2012; Corrado et al., 2018; Park et al., 2017; Lee et al., 2011; Wang et al., 2015; Cao et al., 2015).

Recently, a multicenter phase III prospective, randomised study comparing MI-RH vs O-RH failed to achieve the primary end-point (i.e. non- inferiority of MI-RH in terms of 5-yr disease free survival), due to the documentation of a fourfold increase of hazard ratio for recurrence of disease, leading to the premature closure of the study for safety issues (Ramirez et al., 2018).

These unexpected results rapidly led to contrasting reactions within the scientific community, modifying current ideas regarding the best surgical approach in early-stage cervical cancer and, at the same time, causing still ongoing controversial opinions, including the potential role of gynaecological oncology surgeon (GOS) learning of the minimally invasive approach, which must require specific skills (Kimmig and Ind, 2018; Nezhat et al., 2019; Leitao, 2018; Vergote et al., 2019).

It has been argued that minimally invasive procedures performed by surgeons who are not adequately skilled could have affected survival results of MI-RH (Leitao, 2018). It is surprising that, despite the great interest about the potential impact of the surgeon experience in MI-RH on the oncological outcomes in early-stage cervical cancer, the majority of previous studies evaluated the surgeon learning curve on the basis of the short-term outcomes only (i.e. operative time, complications, conversion rate etc.), or the comparison of survival measures according to time intervals (Yim et al., 2013; Madhuri et al., 2021; Hwang et al., 2012; Oyama et al., 2019; Chong et al., 2009; Cusimano et al., 2019; Liu et al., 2019).

The aim of this study is to investigate whether the surgeon learning curve for MI-RH, evaluated in terms of number of performed procedures, might have an impact on early-stage cervical cancer patient survival.

## Materials and methods

This is a single centre, retrospective study including patients affected by histologically confirmed cervical cancer from Gynaecologic Oncology Units of Fondazione Policlinico-Universitario A. Gemelli-IRCCS, Università Cattolica, Roma. All data were retrospectively collected and then analysed. All women had a histologically confirmed cervical cancer diagnosis and also subscribed a written informed consent about data collection and their processing for scientific purposes. The Institutional Review Board approved the study (IRB number: DIPUSVSP-03-04-2051).

Patients demographics, surgical, pathological, and follow-up data were obtained from an electronic database. Preoperative staging was performed through gynaecologic examination under anaesthesia, pelvic ultrasound, and magnetic resonance imaging. All patients with clinical 2009 FIGO stage (Pecorelli et al., 2009) from IA1 with lymphovascular space invasion to IB1/IIA1 with or without suspicious lymph nodes at preoperative imaging assessment were judged eligible for surgery and included in the study. Exclusion criteria were: neoadjuvant therapy prior to the surgery, completion surgery after an incidental diagnosis of cervical cancer following total or sub-total hysterectomy performed for suspicious benign disease and lack of clinical, pathological or surgical data. Patients treated after December 2018 were excluded from the study because our clinical practice changed after “LACC” trial results (Ramirez et al., 2018).

Patients were primarily treated with radical hysterectomy codified according to the Querleu– Morrow classification (Querleu and Morrow, 2008) and systematic pelvic lymphadenectomy or sentinel lymph node biopsy. Aortic lymphadenectomy was performed according to physician discretion and in cases of intraoperatively assessed metastatic disease involving pelvic lymph nodes as shown at frozen section analysis.

All surgical specimens were evaluated by dedicated pathologists. Adjuvant treatment was recommended according to international guidelines (National Comprehensive Cancer Network Cervical Cancer Guidelines, 2019; Cibula et al., 2018).

### Assessment of gynaecological oncology surgeons experience

The complete learning paths of 11 surgeons were available since all MI-RH procedures for early- stage cervical cancer patients performed during their own careers were traced and recorded. All the 11 surgeons completed their residency in our institution, focusing their training on the field of gynaecologic oncology surgery. Once they became consultants, they performed surgery autonomously. In our institution surgery is generally performed by a consultant as first surgeon, a senior resident as first assistant and a junior resident as second assistant. At least one senior consultant (with more than 10 years of experience as first surgeon) is always available for consultation in case of the less experienced consultant needs.

MI-RH procedures were checked on the basis of the standard guidelines (Cibula et al., 2018). If the surgical data were uncertain or even not complete, the procedures were excluded. Multiple dichotomizations according to the number of MI-RH procedures per GOS were analysed as per survival analyses in order to establish the surgeons learning curve in terms of patient prognosis improvement.

### Statistical Analysis

Disease free survival (DFS) was defined as the time elapsed between surgery and recurrence or date of the last follow-up. Overall survival (OS) has been defined as the time elapsed between surgery and death for cervical cancer or date of the last follow up. The χ2 test or Fisher’s exact test for proportion were used to analyse the distribution of clinical and pathological variables between the two groups. Medians and life tables were computed using the product limit estimate by Kaplan–Meier method (Kaplan and Meier, 1958) and the log-rank test was used to assess the statistical significance (Mantel, 1966). Multiple, sequential Cox regression analyses (Cox, 1972) were conducted in order to calculate the hazard ratios (HRs) according to each increase in number of MI-RHs per surgeon, in order to assess the impact of experience on patient survival improvement. The comprehensive GOS learning curve was assessed considering the progressive improvement on patients’ survival and described according to the formula: ∑10^(1/HR) . Cox regression model with stepwise variable selection (Cox, 1972) was used to analyze the impact of experience, based on the number of performed procedures and on the role of different parameters as prognostic factors for DFS. Statistical Package for Social Sciences software version 25.0 (IBM Corporation, Armonk, NY, USA) was adopted to carry out all statistical calculations. For all analyses a p value < 0.05 was considered significant.

## Results

**Figure 1:**
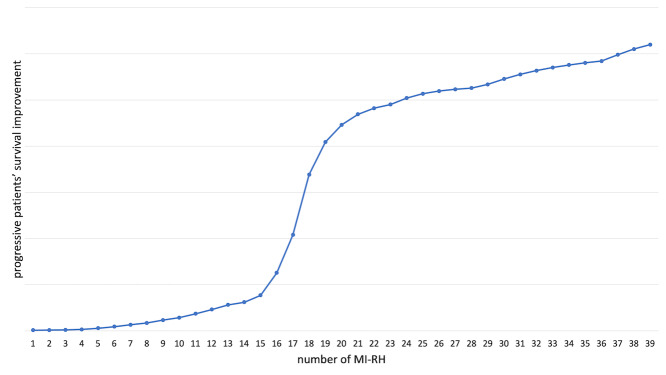
Comprehensive gynaecological oncology surgeon (GOS) learning curve —Trend of the learning curve was calculated considering the cumulative sum of progressive improvement on patients’ survival according to the increase of number performed by a GOS during his/her career.

**Figure 2:**
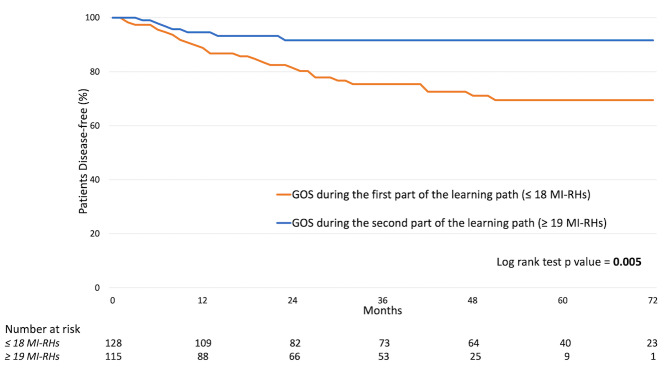
Disease-free survival curve according to gynaecological oncology surgeon (GOS) experience.

From January 1998 to December 2018, 685 consecutive cases of early-stage cervical cancer patients were retrieved from the electronic database. We began to implement the minimally invasive approach in our clinical practice for cervical cancer from 2010 and from 2012 it became the most used. An increase in the rate of patients treated with open surgery was recorded in 2018 (Figure 1). From the whole series of 685 patients, 300 were excluded because they had been managed by the open approach, leaving 385 patients treated with a minimally invasive procedure. Nineteen patients were excluded due to completion surgery after total or subtotal hysterectomy performed for suspicious benign disease, and 3 were excluded due to lack of surgical data, thus leaving 363 cases (Figure 2).

Two hundred and forty-three were treated by surgeons who completed all their learning path in our institution, while 120 were treated by external consultants.

Main analyses were conducted on these 243 cases with complete pathological and surgical information (Table I).

**Table I:** Surgical and clinical-pathological characteristics of the series.

Characteristics	N. (%)
Patients	243
Median Age, yrs (range)	47 (25-80)
Radicality of Surgery^a^	
- Type A	43 (17.7)
- Type B	111 (45.7)
- Type C	89 (36.6)
Lymph Node Surgery	
- Sentinel Lymph Node Biopsy	47 (19.3)
- Pelvic Lymphadenectomy	196 (80.7)
Aortic Lymphadenectomy	
- No	159 (65.4)
- Yes	84 (34.6)
Pathological FIGO stage	
- IA1	52 (21.4)
- IA2	15 (6.2)
- IB1	131 (53.9)
- IB2	8 (3.3)
- IIA1	9 (3.7)
- IIA2	5 (2.1)
- IIB	23 (9.5)
Histology	
- Squamous carcinoma	157 (64.6)
- Other	86 (35.4)
Grading	
- G1 / G2	178 (73.3)
- G3	53 (21.8)
Tumor diameter	
- ≤ 20 mm	157 (64.6)
- > 20 mm	86 (35.4)
Lymphovascular space invasion	
- Absent	154 (63.4)
- Present	89 (36.6)
Lymph node status	
- Negative	198 (81.5)
- Positive	45 (18.5)
Adjuvant therapy	
- No	140 (57.6)
- Radiotherapy	46 (18.9)
- Chemotherapy	4 (1.6)
- concomitant chemoradiotherapy	53 (21.8)

^a^according to Querleu and Morrow classifications

Median age was 47 years (range 25-80). Most patients underwent type B hysterectomy according to Querleu-Morrow classification. One hundred ninety-six (80.7%) underwent systematic pelvic lymphadenectomy while 47 (19.3%) underwent sentinel lymph node biopsy. Aortic lymphadenectomy was performed in 84 (34.6%) cases. The majority of patients had a pathological FIGO stage IB (139, 57.2%), a squamous histotype (157, 64.6%) and a tumor diameter < 2 cm (157, 64.6%). Adjuvant therapy was performed in 103 (42.3%) cases.

As of September 2019, the median follow-up was 38 months (range: 3-111); 35 (14.4%) patients relapsed and 11 (4.5%) died from disease.

Surgeons learning path was retraced classifying patients according to the progressive number of MI-RH for early-stage cervical cancer performed by each GOS (i.e. the first patients operated by each surgeon were classified with number 1, the second were classified with number 2 and so on).

Not all surgeons started their career as first operator at the same time and 6 of them became consultants after 2010.

The 11 surgeons included in the analysis performed a median of 17 MI-RHs for early-stage cervical cancer (range: 6–47). Four of them (36.3%) performed more than 30 procedures as first surgeon. The mean number of MI-RHs performed for early- stage cervical cancer per year by each surgeon was 6.2 (range: 2.8 – 7.1).

Multiple, sequential survival analyses were conducted according to the number of MI-RHs per GOS in order to assess the impact of experience on patient survival improvement (Figure 3). Excluding the range of the first 4 procedures, each unitary increment in MI-RHs performed by a surgeon provided a reduction of patients’ risk of recurrence (hazard ratio: from 0.974 to 0.321).

In particular, the experience, measured by evaluating the number of performed procedures, showed a statistically significant impact on patients’ prognosis starting from the 14th MI-RH performed (hazard ratio: 0.499; 95%CI: 0.251-0.992; p value= 0.048). This statistical significance lasted until the 21st MI-RH performed (hazard ratio: 0.424; 95%CI: 0.185-0.974; p value=0.043). The peak of reduction of the recurrence risk was shown when the surgeon performed the 19th MI-RH (hazard ratio: 0.321; 95%CI: 0.140-0.737; p value=0.007). Data details are shown in Table II.

**Table II:** Cox regression analyses association between gynaecological oncology surgeon (GOS) experience, defined by sequential dichotomizations of the number of MI-RS performed by the GOS, and disease-free survival.

Number of MI-RH performed	Hazard Ratio	95% CI*	p value
≤ 1 vs ≥ 2	0.891	0.215-3.373	0.880
≤ 2 vs ≥ 3	1.968	0.472-8.202	0.353
≤ 3 vs ≥ 4	1.439	0.508-4.078	0.494
≤ 4 vs ≥ 5	0.974	0.425-2.231	0.950
≤ 5 vs ≥ 6	0.705	0.338-1.468	0.350
≤ 6 vs ≥ 7	0.665	0.331-1.337	0.252
≤ 7 vs ≥ 8	0.615	0.312-1.211	0.160
≤ 8 vs ≥ 9	0.632	0.323-1.236	0.180
≤ 9 vs ≥ 10	0.558	0.287-1.085	0.086
≤ 10 vs ≥ 11	0.581	0.299-1.130	0.110
≤ 11 vs ≥ 12	0.518	0.265-1.012	0.054
≤ 12 vs ≥ 13	0.511	0.260-1.007	0.052
≤ 13 vs ≥ 14	0.499	0.251-0.992	**0.048**
≤ 14 vs ≥ 15	0.568	0.286-1.131	0.107
≤ 15 vs ≥ 16	0.460	0.225-0.941	**0.033**
≤ 16 vs ≥ 17	0.372	0.174-0.796	**0.011**
≤ 17 vs ≥ 18	0.343	0.156-0.756	**0.008**
≤ 18 vs ≥ 19	0.321	0.140-0.737	**0.007**
≤ 19 vs ≥ 20	0.351	0.153-0.805	**0.013**
≤ 20 vs ≥ 21	0.389	0.169-0.893	**0.026**
≤ 21 vs ≥ 22	0.424	0.185-0.974	**0.043**
≤ 22 vs ≥ 23	0.471	0.205-1.083	0.076
≤ 23 vs ≥ 24	0.526	0.229-1.210	0.131
≤ 24 vs ≥ 25	0.466	0.193-1.128	0.090
≤ 26 vs ≥ 27	0.508	0.210-1.231	0.134
≤ 26 vs ≥ 27	0.567	0.234-1.375	0.210
≤ 27 vs ≥ 28	0.634	0.262-1.538	0.314
≤ 28 vs ≥ 29	0.697	0.287-1691	0.425
≤ 29 vs ≥ 30	0.528	0.310-1.823	0.528
≤ 30 vs ≥ 31	0.481	0.169-1.368	0.170
≤ 31 vs ≥ 32	0.502	0.176-1.1430	0.197
≤ 32 vs ≥ 33	0.524	0.184-1.495	0.227
≤ 33 vs ≥ 34	0.547	0.192-1.562	0.260
≤ 34 vs ≥ 35	0.573	0.201-1.639	0.299
≤ 35 vs ≥ 36	0.602	0.210-1.723	0.334
≤ 36 vs ≥ 37	0.633	0.221-1.814	0.633
≤ 37 vs ≥ 38	0.468	0.142-1.542	0.212
≤ 38 vs ≥ 39	0.479	0.145-1.581	0.227
≤ 39 vs ≥ 40	0.503	0.152-1.659	0.259

Bold values indicate statistically significant; *CI: confidence interval

The comprehensive GOS learning curve showed only a very small impact of the experience on patients’ prognosis during the first procedures (Figure 1). A substantial and steep improvement of patients DFS was shown between the 14^th^ and the 21^st^ performed MI-RHs. Subsequently, as the number of performed MI-RHs increased, a stable, small effect on the patient prognosis was observed.

According to these results, patients were divided in two groups regarding the operators experience at the time of surgery. The first one included patients managed by a gynaecologic oncologist at the beginning of the learning path in MI-RH for early- stage cervical cancer (<18 procedure performed), while the other group included patients treated by a physician who gained enough experience (>19MI-RH performed).

There was no difference between the two groups according to the main pathological risk factors and the adjuvant treatment administered (Table III).

**Table III:** Clinical-pathological characteristics of patients grouped according to gynaecological oncology surgeon (GOS) experience at the time of their surgery.

	GOS during the first part of the learning path (< 18 MI-RH)	GOS during the second part of the learning path (> 19 MI-RH)	
Characteristics	N. (%)	N. (%)	p Value
Patients	128	115	243
Pathological FIGO stage			
- IA1	31 (24.2)	21 (18.3)	
- IA2	11 (8.6)	4 (3.5)	
- IB1	65 (50.8)	66 (57.4)	
- IB2	2 (1.6)	6 (5.2)	
- IIA1	3 (2.3)	6 (5.2)	
- IIA2	4 (3.1)	1 (0.9)	
- IIB	12 (9.4)	11 (9.6)	0.154
Histology			
- Squamous carcinoma	86 (67.2)	71 (61.7)	
- Other	42 (32.8)	44 (38.3)	0.375
Grading			
- G1 / G2	97 (75.8)	90 (78.3)	
- G3	31 (24.2)	25 (21.7)	0.678
Tumor diameter			
- ≤ 20 mm	86 (67.2)	73 (63.5)	
- > 20 mm	42 (32.8)	42 (36.5)	0.540
Lymphovascular space invasion			
- Absent	79 (61.7)	75 (65.2)	
- Present	49 (38.3)	40 (34.8)	0.850
Lymph node status			
- Negative	102 (79.7)	96 (83.5)	
- Positive	26 (20.3)	19 (16.5)	0.448
Adjuvant therapy			
- No	77 (60.2)	66 (57.4)	
- Radiotherapy	28 (21.9)	15 (13.0)	
- Chemotherapy	2 (1.6)	2 (1.7)	
- concomitant chemoradiotherapy	21 (16.4)	32 (27.8)	0.171

The survival outcomes of patients treated by a gynaecologic oncologist once he/she had gained experience in MI-RH were significantly better than those of patients treated at the beginning of the learning path (3-years DFS: 91.6% vs 75.4% respectively, p= 0.005, Figure 2). Between the two periods of the learning curve there was a non- statistically significant difference in terms of patient OS (3-years OS: 96.7% vs 91.3%respectively, p=0.121, data not shown).

Univariate analyses showed that GOS practice in MI-RH was significantly associated to patient DFS. Multivariate analyses confirmed that the GOS experience resulted as an independent factor on patient prognosis (Table IV).

**Table IV:** Cox’s regression uni- and multi-variate analyses of pathologic and surgical parameters associated to disease-free survival.

	Univariate Analysis		Multivariate Analysis	
Characteristics	HR (95% CI) *	p Value	ß	p Value
Pathological FIGO stage	1.444 (1.004 – 2.078)	**0.048**	/	0.651
Histology	1.075 (0.534 – 2.162)	0.840	/	/
Grading	1.402 (0.687 – 2.863)	0.354	/	/
Tumor diameter	4.164 (2.055 – 8.438)	**0.001**	1.356	**0.001**
Lymphovascular space invasion	1.560 (1.199 – 2.030)	**0.002**	/	0.120
Lymph nodal status	1.267 (1.003 – 2.268)	**0.047**	/	0.202
GOS experience (< 18 vs > 19 MI-RH)	0.321 (0.140 – 0.737)	**0.007**	-1.013	**0.018**

* HR: Hazzard Ratio, CI: confidence interval; Bold values indicate statistically significant

A supplementary analysis conducted on the 171 patients managed by the laparoscopic approach only confirmed that a gynaecologic oncologist significantly influences patients prognosis according to the level of experience (3- years DFS of “GOS during the first part of the learning path” vs. “GOS during the second part of the learning path”: 93.4% vs 75.5%, p=0.013, Figure 4).

Figure 5 shows that the GOS learning curve had a relatively relevance in cases with <2 cm tumor size (3- years DFS of “GOS during the first part of the learning path” vs. “GOS during the second part of the learning path”: 94.2% vs 87.6%, p value=0.209). On the other hand, the GOS experience was statistically relevant when the tumor dimension was >2 cm (3-years DFS of “GOS during the first part of the learning path” vs. “GOS during the second part of the learning path”: 86.4% vs 56.8%, p=0.017).

## Discussion

This study demonstrates that there is a strict relationship between GOS experience and early- stage cervical cancer patient prognosis based on the number of MI-RH procedures and that it is independent of other, well known, prognostic factors of cervical cancer (Fagotti et al., 2016; Wagner et al., 2013; Pedone Anchora et al., 2020a). In the vast majority of previous studies concerning the learning curve of surgeons, their experience has been evaluated in terms of peri-operative outcomes according to the absolute number of radical hysterectomies (Yim et al., 2013; Madhuri et al., 2021; Hwang et al., 2012; Oyama et al., 2019; Chong et al., 2009; Cusimano et al., 2019; Liu et al., 2019).

The present analysis showed that the increase of GOS experience allowed better results in terms of oncological outcomes. Furthermore, surgeon experience affected patient prognosis following the typical trend of a learning curve. At the beginning, a steady recurrence-risk reduction trend was associated with the increase of number of MI-RHs performed. Subsequently, there was a phase of steep growth of the curve. This could be considered the most significant period of the learning process since each procedure performed improves the surgeon outcomes and has a strong impact on the patient prognosis. Lastly, once a surgeon achieved a certain level of experience, further practice gave only a small and stable improvement on patient survival.

In addition, we showed that the survival related results in the laparoscopic group were in accordance with the primary analysis. On the other hand, the sample of patients treated with the robotic approach was too small to draw any relevant conclusion (data not shown). The experience appeared as having a greater impact among high-risk cases, since the difference between the different phases of a learning curve was significant especially in cases of tumor > 2 cm.

In our series the peak of recurrence risk reduction was represented by 19th MI-RHs. One could argue that the identified cut-off could not be generalised to other series due to the limitations inherent to a retrospective study (i.e.selection bias, missing data/variables, etc.), and even to a single-centre patient series. We had planned at the beginning to exclude in the present analysis other institutions because the homogeneity of surgical procedures, equipment/facilities and the standardised selection criteria for recruitment shared by all surgeons could be better guaranteed by a single-institution series.

As far as the identified cut-off, we acknowledge that the absolute number of performed procedures without the referral to a specific time interval could have introduced a bias in the evaluation of the surgeon skillfulness. However, although we could have had details relative, for instance, to the number of MI-RHs performed by per month, this ratio would have not been sufficiently reliable given the heterogeneity of time frames and modalities of individual learning path.

Moreover, in the present series, we failed to identify a significant difference in terms of OS according to surgeon learning path. However, one could consider that, while DFS could be strictly related to basic treatment and pathological tumor features, the OS may also be influenced by other factors such as the site (Quinn et al., 2006; Boussios et al., 2016) and management of recurrence (Dornhöfer and Höckel, 2008; Gadducci et al., 2010; Vizzielli et al., 2017; Suzuki et al., 2019).

Present findings should be evaluated in the current clinical context. After publication of the LACC trial, some international guidelines and statements of the Scientific Societies generally accept and recommend implementing the trial evidence in clinical practice (NCCN Cervical Cancer Guidelines, 2019; Hillemanns et al., 2019) while others suggest considering its results along with those of other publications (Querleu et al., 2020, Yim et al. 2019). Despite different opinions and statements from experts and Societies, all of them stress the importance of providing accurate counseling to patients regarding advantages and disadvantages along with all factors that could influence outcomes of each surgical approach.

Literature is constantly enriched with studies comparing MIS to open surgery for early-stage cervical cancer (Brandt et al., 2020) also in specific subgroups (Pedone Anchora et al., 2020b) and with research that investigates possible explanation of LACC trial results (Casarin et al., 2020). This is not the topic of the present study. The concept that not all surgeons who perform MI-RH have similar oncological outcomes should be taken into account in future studies comparing open and minimally invasive surgery for early-stage cervical cancer (NCT03739944, NCT03719547).

Recently, the European Society of Gynecological Oncology Group developed quality indicators for surgical treatment of cervical cancer (Cibula et al., 2020). It is recommended that patients are treated in high-volume centres with specialised surgeons. Present findings underline that not all the surgeons in a high-volume hospital have the same proficiency in all settings and that the minimally invasive treatment for early-stage cervical cancer requires a very specific training.

The main conclusion of the present study is that the experience that a gynaecological oncologist can achieve by practice in MI-RH is an independent prognostic factor for early-stage cervical cancer patients.

Although other non-measurable data, such as the individual comprehensive surgical skill and the competence regarding the disease, the pelvic anatomy etc., may influence the GOS skillfulness, a disease-specific training under the supervision of an expert surgeon should be required prior to perform MI-RH safely in cases of early-stage cervical cancer.

A prospective trial is advisable to confirm our findings and we strongly believe that these results could encourage Scientific Societies to define better the minimum level of training required for gynaecologic oncologic surgeons, not only in the field of early-stage cervical cancer, in order to provide high, standardised care to our patients.
